# Ansofaxine Hydrochloride inhibits hepatocellular carcinoma growth and enhances targeted therapy through the EGFR/MAPK pathway

**DOI:** 10.3389/fonc.2025.1523570

**Published:** 2025-07-30

**Authors:** Yongfei He, Qiang Tao, Shutian Mo, Meifeng Chen, Jicai Wang, Hang Zhai, Shengjie Hong, Qiang Gao, Guangquan Zhang, Chuangye Han, Xianjie Shi

**Affiliations:** ^1^ Department of Hepatobiliary and Pancreatic Surgery, The Eighth Affiliated Hospital of Sun Yat-sen University, Shenzhen, Guangdong, China; ^2^ Department of Hepatobiliary Surgery, the First Affiliated Hospital of Guangxi Medical University, Nanning, China; ^3^ Department of Hepatobiliary Surgery, The Affiliated Hospital of Inner Mongolila Medical University, Huhehaote, China

**Keywords:** hepatocellular carcinoma, Ansofaxine Hydrochloride, network pharmacology, EGFR/MAPK pathway, depression

## Abstract

**Background:**

Hepatocellular carcinoma (HCC) is a common tumor that endangers health. Depression will affect the therapeutic effect of HCC, and depression and HCC promote and influence each other. Ansofaxine Hydrochloride is a novel antidepressant, and its anti-HCC effect remains to be confirmed.

**Objectives:**

This study aimed to investigate the effect of Ansofaxine Hydrochloride on HCC and its molecular mechanism.

**Methods:**

The potential targets and signaling pathways of Ansofaxine Hydrochloride against HCC were obtained by network pharmacology, and the key targets were explored by molecular docking techniques. Hepatocellular carcinoma cells were treated with different concentrations of Ansofaxine Hydrochloride, and the effects of Ansofaxine Hydrochloride on the biological function of hepatocellular carcinoma cells were evaluated by CCK8, migration, invasion, and clonal formation tests. Subsequently, a subcutaneous hepatocellular carcinoma mouse model was established to evaluate the effect of Ansofaxine Hydrochloride on the growth of hepatocellular carcinoma tissue *in vivo*, and an enzym-linked immunosorbent assay was used to detect the levels of dopamine (DA) and 5-hydroxytryptamine (5-HT) in peripheral blood. HE and immunohistochemical staining were used to detect the pathological changes of tumor tissue and the types and proportions of macrophages. Finally, the expression levels of key genes in the EGFR/MAPK pathway were detected by Reverse Transcription Real-time Quantitative analysis.

**Results:**

There are 87 common drug-disease targets between Ansofaxine Hydrochloride and HCC, including EGFR, GRB2, and SRC, which are mainly involved in EGFR, MAPK, and PI3K/AKT signaling pathways. Molecular docking showed that Ansofaxine Hydrochloride has good binding activity to EGFR, GRB2, and other key targets. *In vitro* experiments showed that Ansofaxine Hydrochloride has significant inhibitory effects on proliferation, migration, invasion, and clonal formation of HCC cells. *In vivo* experiments showed that Ansofaxine Hydrochloride has a synergistic effect of enhancingLenvatinib anti-HCC, enhancing peripheral blood DA level, promoting M1 macrophage infiltration, and enhancing immune anti-tumor effects, and is associated with the reduction of EGFR/MAPK pathway-related genes.

**Conclusion:**

Our study suggests that Ansofaxine Hydrochloride has anti-HCC and immunomodulatory effects, with the EGFR/MAPK pathway as a potential key mechanism of action.

## 1 Introduction

Hepatocellular carcinoma (HCC) is the third leading cancer-related cause of death worldwide, causing hundreds of thousands of deaths each year and posing a serious public health challenge (1, 2). At the same time, patients’ mental health problems have also received more and more attention, and the incidence of depression in cancer patients is significantly higher than that in patients with other diseases (3). Studies have shown that depression is the most common psychological problem in patients with hepatocellular carcinoma after surgery, resulting in reduced treatment compliance of patients and seriously affecting prognosis (4). Existing evidence points out that depression promotes the progression of various tumors through the neuroimmune system and affects the therapeutic effect of tumors (5–7). Therefore, there is great clinical value in developing drugs that can treat both tumors and depression.

At present, early hepatocellular carcinoma is often treated with surgery, including hepatectomy and liver transplantation, which can effectively remove tumor tissue. For patients who are not eligible for surgery or are in advanced stages, interventional treatments such as transcatheter chemoembolization and radiofrequency ablation are available as options to control tumor development locally (8). In recent years, systemic therapy with targeted immunotherapy and local therapy has achieved remarkable results (9, 10). Since the approval of small molecule tyrosine kinase inhibitors (TKI) by the U.S. Food and Drug Administration in 2001, targeted drugs with higher potency and lower toxicity have ushered in a golden age of development.Lenvatinib is an oral small-molecule TKI similar to sorafenib.Lenvatinib plays a key role in the treatment of middle and advanced stages and prevention of HCC recurrence, but its efficacy is still poor, mainly related to the resistance ofLenvatinib (11, 12). The drug combination is an effective way to solve drug resistance (13). Therefore, it is necessary to develop new drugs to enhance the efficacy ofLenvatinib while reducing resistance synergistically.

Toxic side effects and drug resistance caused by targeted immunotherapy in anti-tumor treatments can hurt treatment. The combined use of a variety of anti-tumor drugs can induce a synergistic effect and reduce or delay the generation of drug resistance and other adverse factors to improve the overall therapeutic effect. Existing studies show that approximately one quarter of HCC patients suffer from depression. There is a significant association between depression and liver cancer. Depressive mood may increase the risk of liver cancer and accelerate the progression of the disease through multiple mechanisms. At the same time, depression is highly prevalent among liver cancer patients and forms a vicious cycle, requiring comprehensive intervention measures (4). Some studies have found that the combination of antidepressants and immune checkpoint inhibitors can enhance the anti-tumor immune effect of the body while treating depression (14, 15). Monoamine oxidase inhibitors and PD-1 therapy can inhibit the differentiation of macrophages into M2 while inhibiting tumors (16). Some scholars have also carried out relevant studies on antidepressants in hepatocellular carcinoma. Escitalopram oxalate, a therapeutic drug for major depressive disorder, has anticancer potential and reduces the risk associated with HCC by inducing autophagy to inhibit the proliferation of hepatocellular carcinoma cells (17, 18). Ansofaxine Hydrochloride is a novel anti-weight depression drug, and phase III clinical trials have shown that it is well tolerated (19). Preliminary studies have confirmed that AH can increase the proportion of natural killer cells (NKs) and M1 macrophages in tumor tissues, increase peripheral dopamine levels, and reduce serotonin uptake. Combined treatment with AH may enhance the efficacy of tumor immunotherapy for colon cancer (20). However, Ansofaxine Hydrochloride has not been studied in the treatment of hepatocellular carcinoma. Therefore, we used Ansofaxine Hydrochloride as an entry point to verify its inhibitory potential against HCC *in vitro* and *in vivo* and to initially elucidate its mechanism of action.

## 2 Materials and methods

### 2.1 Experimental material

Huh7 and Hepa1–6 cells and special media were purchased from Procell (China) and cultured at 37°C and 5%CO_2_. Ansofaxine hydrochloride (Cat No.HY-U00096, MCE), Lenvatinib (Cat No.HY-10981, MCE), DA(Dopamine) ELISA Kit (Cat No.D751019, Sangon Biotech), ST/5-HT ELISA Kit (Cat No.D751013, Sangon Biotech), CD86 (Cat No. 83523-4-RR, Proteintech), CD206 (Cat No. 18704-1-AP, Proteintech), Anti-Ki67 Mouse mAb (Cat No. GB121141-100, Servicebio), RP-conjugated Goat Anti-Rabbit IgG(H+L) (Cat No. SA00001-2, Proteintech), HRP-conjugated Goat Anti-Mouse IgG(H+L) (Cat No. SA00001-1, Proteintech), SteadyPure General-purpose RNA Extraction Kit II (AG21022, Steadypure) Accurate Biology), SYBR Green Pro Taq HS Pre-mixed qPCR Kit (AG11702, Accurate Biology), Hematoxylin-Eosin (HE) Stain Kit (G1120, Solarbio), CCK8 solution (BS350B, Biosharp).

### 2.2 Network pharmacology

#### 2.2.1Target screening of drugs and diseases

Use the PubChem database (https://pubchem.ncbi.nlm.nih.gov/) to obtain Ansofaxine hydrochloride structure. Import the PharmMapper database (http://www.lilab-ecust.cn/pharmmapper/) the Swiss Target Prediction database (http://www.swisstargetprediction.ch/) and the SEA database (https://sea.bkslab.org/) to obtain related targets. The unique target is obtained after the target name is corrected and unified through the UniProt database (https://www.uniprot.org/) and deduplication. With “Hepatocellular carcinoma” as the keyword in the OMIM database (https://omim.org/) and Genecards database retrieval (https://www.genecards.org/), we are to go after the heavy disease targets. In Venny2.1 online software drawing tools platform (https://bioinfogp.cnb.csic.es//tools/venny) on import Ansofaxine targets and Hepatocellular hydrochloride The target of carcinoma is drawn by Wayne diagram, and the common target of drug and disease is obtained after the intersection of the two.

#### 2.2.2 PPI network construction and core target analysis

The common targets of Ansofaxine hydrochloride-Hepatocellular carcinoma are imported into the STRING database (https://string-db.org/) for search. Set the protein type as “Homo sapiens” and the minimum interaction threshold as 0.9. The network relationship data of target interactions were obtained and imported into Cytoscape software to map the protein interaction network. The size and color of a node vary according to the degree value of the node. Import the PPI network to Cytoscape 3.9.1, use NetworkAnalyzer to perform topology analysis, and select core targets based on the degree value. A target with a higher degree is more important.

#### 2.2.3 Enrichment analysis

Use a database of David (https://david.ncifcrf.gov/) to GO and KEGG enrichment analysis of common targets, and the enrichment results are visualized using R language, draw a bar chart bubble chart.

### 2.3 Molecular docking

Compounds from PubChem database (https://pubchem.ncbi.nlm.nih.gov/) download SDF format, import the ChemDraw in 3D, to minimize energy use was module, acquiring the advantage of the lowest energy conformation and save as mol2 files, AutodockTools1.5.6 was used to hydrogenate the ligand, charge it, detect the root of the ligand, search and define the rotable bond, and save it as pdbqt file. The 3D structure of the protein was downloaded from the RCSB PDB database (www.rcsb.org). PYMOL was used to remove water molecules and small molecules from the protein, then hydrogen atoms were added by AutodockTools1.5.6, and Gasteiger charge was calculated. Define it as a receptor and save it as a pdbqt file. The ligands were docked to the receptors using Autodock vina 1.1.2, and the binding patterns were analyzed and visualized with PYMOL.

### 2.4 *In vitro* and *in vivo* experiments

#### 2.4.1 CCK8 detection

A total of 5×10^3^ Huh7 and Hepa1–6 cells were inoculated in 96-well plates and cultured for 24 hours. Subsequently, different concentrations of Ansofaxine hydrochloride were added by changing the medium. After 24 hours of intervention, 10μL of CCK8 solution was added to each well and incubated at 37°C for another 2 hours. Absorbance (OD) was read at 450 nm using an enzyme-labeler and cell inhibition rates were calculated. Cell inhibition rate (%) = (control group OD- administration group OD)/(control group OD- blank group OD) %.

#### 2.4.2 Migration and invasion experiments

Huh7 and Hepa1–6 cells were divided into 2 groups and treated with different concentrations of AH for 24 hours. Matrigel was diluted in serum-free medium in 280ug/ml diluted Transwell chamber (aperture 8μm, Falcon) and incubated at 37°C for 60 min for gel polymerization. Cells were collected and resuspended in a serum-free medium. Then, 300µL serum-free medium containing 3×10^4^ cells was added to the Transwell, and 700µL medium containing 10% FBS was added to the inferior cavity. The samples were cultured in the incubator for 48 hours, fixed with 4% paraformaldehyde for 30 minutes, and washed twice with PBS. Then add 1% crystal violet to dye for 30 minutes, wash with PBS and residual dye solution. The cells in the upper chamber were gently swabbed with a cotton swab and photographed under a microscope. The bottom surface of the cavity was eluted with 30% acetic acid 300 μL. Add about 100 μL solution to each hole of the 96-well plate. OD values at 590nm were read with an enzyme label.

#### 2.4.3 Cell cloning

Huh7 and Hepa1–6 cells were collected and divided into two groups. Cells were added with corresponding concentrations of AH and cultured for 24 hours. Cell counts were collected separately for each group. Six well plates were inoculated with 500 cells/Wells and cultured for 2 weeks until colony formation. The cells were fixed with 4% paraformaldehyde for 30 minutes, washed twice with PBS, and stained with 1% crystal violet for 20 minutes. Colony formation was observed under an inverted microscope and clone formation rate was calculated.

#### 2.4.4 Establishment and administration of animal models

C57BL/6 mouse (male, 4–5 weeks old, 18–22 g) was purchased from the Animal Experimental Center of Guangxi Medical University. All experiments were approved by the Animal Care and Utilization Committee of the First Affiliated Hospital of Guangxi Medical University (No. 2024-E782-01). Animal ethics review shall be conducted concerning the Guidelines for the Treatment of Experimental Animals of the Ministry of Science and Technology of the People’s Republic of China and the national standard GB/T35892–2018 Guidelines for the Ethical Review of Experimental Animals - Animal Welfare of the People’s Republic of China. A large number of Hepa1–6 cells were cultured and 1×10^6^ Hepa1–6 cell suspension was injected subcutaneously into the left axilla of mice to establish a mouse hepatocellular carcinoma model. After tumor growth to 100 mm3, the mice were randomly divided into 4 groups: model group, Ansofaxine hydrochloride group, lenvastinib group, and combined drug group. According to the experimental groups, the model group was given intraperitoneal injection of normal saline (50mg/kg) + intragastric injection of normal saline (10mg/kg). The Ansofaxine hydrochloride group was intraperitoneally injected with Ansofaxine hydrochloride (50mg/kg) + saline hydrochloride (10mg/kg), and theLenvatinib group was intraperitoneally injected with saline (50mg/kg) +Lenvatinib hydrochloride (10mg/kg). Combined group Ansofaxine hydrochloride is injected intraperitoneally (50mg/kg) andLenvatinib hydrochloride is orally administered (10mg/kg), 3 pills in each group. After 7 days of continuous treatment, the tumor growth was monitored every other day for 7 days. The tumor volume was calculated as (width 2×length)/2.

#### 2.4.5 ELISA was used to detect DA and 5-HT levels

Follow the ELISA kit instructions for DA and 5-HT. The concentration gradient is set with the standard substance, the standard curve is drawn according to the measured OD value, and the corresponding concentration is found on the coordinate according to the light absorption value of the sample.

#### 2.4.6 RT-qPCR analysis

The SteadyPure Universal RNA Extraction Kit II (Accurate Biology, China) was used to isolate total RNA from Huh and Hepa1–6 cells. The RNA was reverse-transcribed into cDNA using the SYBR Green Pro Taq HS premixed qPCR kit, and then the mRNA expression level of related genes was detected by QuantStudio 6 Flex real-time fluorescent quantitative PCR system according to the product experimental protocol. Gene expression (F) =2-△△Ct, △△Ct= (Ct target gene -Ct internal reference) experimental group - (Ct target gene -Ct internal reference) control group. The experiment was repeated three times. The primer sequence is in **Table 1**.

**Table 1:** Primer sequences for RT-qPCR.

Gene		Forward primer (5′–3′)	Reverse primer (5′–3′)
Human	*EGFR*	CTGGGTGCGGAAGAGAAAGAATA	CCAAAGGTCATCAACTCCCAAAC
	*RAS*	AGTGCCTTGACGATACAGCTAAT	TCCTCATGTACTGGTCCCTCATT
	*MEK*	GGAGAGCATTGAGATTGACCAGA	CCAAGTTCTCCAGGTCGTTGATT
	*ERK*	GTGTTGCAGATCCAGACCATGAT	TGCAGCCTACAGACCAAATATCA
	*GRB2*	CCTGGACTTAGCATTGTGAG	TTATCATCAGCAGGGAGAG
	*ACTB*	CTCCTTAATGTCACGCACGAT	CATGTACGTTGCTATCCAGGC
Mouse	*Egfr*	GCCACGCCAACTGTACCTAT	CACTGCCATTGAACGTACCC
	*Ras*	AAAGAGTGCCCTGACCATCC	CCCCATCAATGACCACCTGT
	*Mek*	TGGGGGTACGCTGAGACATC	ATCTTGTCCCACTTTCCAGGC
	*Erk*	TCCAACCTCCTGCTGAACAC	ATCTGGATCTGCAACACGGG
	*Grb2*	GGTTGCTCTGTTGCTTCTGC	CACACAATGCCACCCGTGA
	*Actb*	GCCGGACTCATCGTACTCC	GTGACGTTGACATCCGTAAAGA

#### 2.4.7 HE staining and immunohistochemistry

Some mouse tumors were fixed in formaldehyde for 24h, dehydrated, waxed, and sliced. The sections of each group were dewaxed and stained with hematoxylin-eosin to observe the pathological changes in tissue structure. Sections were dewaxed, antigens repaired with a citric acid solution, and incubated with H2O2. Sections were sealed with non-immunoreactive serum and incubated overnight with anti-KI67, CD86, and CD206 antibodies. Sections were incubated with secondary antibodies, and stained with DAB, and hematoxylin was retained. The sections were observed and analyzed with an optical microscope.

### 2.5 Statistical analysis

R version 4.4.1 was used for data analysis. T-test analysis was performed for two groups of samples and one-way ANOVA was performed for multiple groups of samples. P<0.05 indicated that the difference between the data was statistically significant.

## 3 Results

### 2.1 Ansofaxine hydrochloride target prediction

According to the chemical structure of Ansofaxine hydrochloride, relevant targets were obtained by importing the PharmMapper database, Swiss Target Prediction database, and SEA database, respectively. A total of 299 targets, such as CDK6, STAT1, and MAPK14, were obtained after the target names were corrected and unified by the UniProt database and duplicated.

### 3.2 Screening of common targets of Ansofaxine hydrochloride and Hepatocellular carcinoma

**Figure 1:**
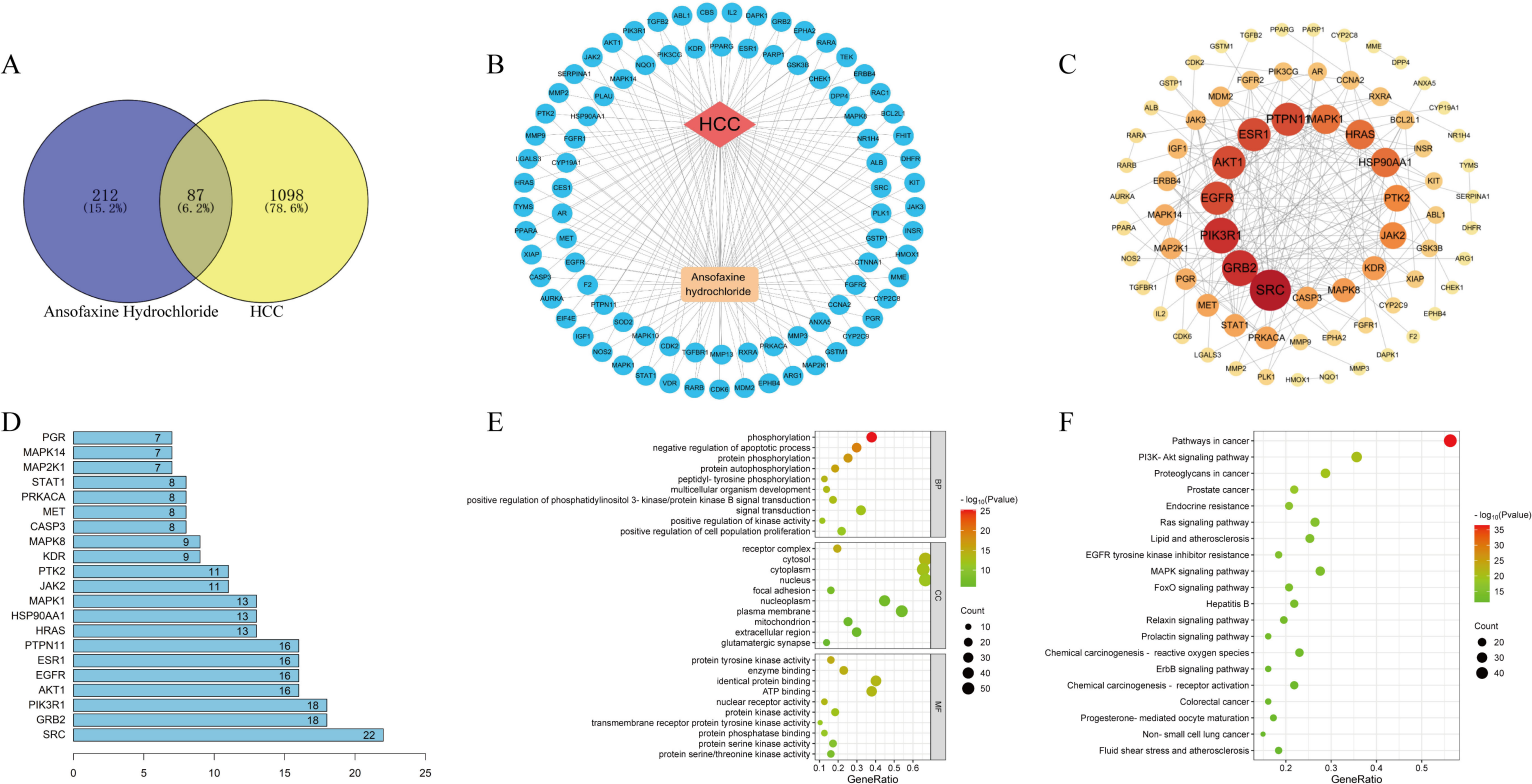
Network pharmacological analysis of Ansofaxine hydrochloride on hepatocellular carcinoma. **(A)** Wayne diagram of intersection genes of Ansofaxine hydrochloride and hepatocellular carcinoma. **(B)** Disease-target-component network diagram. **(C)** Protein interaction network. **(D)** Core target ranking based on PPI topological analysis. GO analysis and KEGG analysis of **(E, F)** core target.

Hepatocellular carcinoma was searched in OMIM and Genecards databases, and 1185 disease targets were obtained after weight removal. A total of 299 drug targets and 1185 disease targets were imported into the Venny2.1 online software mapping tool platform to draw the Wayne diagram. 87 drug-disease common targets such as CYP19A1, HSP90AA1 and EGFR (**Figures 1A, B**) were obtained after their intersection.

### 3.3 PPI network construction and core target analysis

To further explore the core targets of the interaction between Ansofaxine hydrochloride and Hepatocellular carcinoma, 87 common targets were imported into the STRING database to construct a PPI network. Topological analysis was carried out by the NetworkAnalyzer tool of Cytoscape 3.9.1, and the top 20 core genes such as SRC, GRB2, and EGFR (**Figures 1C, D**) were obtained.

### 3.4 Enrichment analysis

The 87 common targets were enriched and analyzed by David database GO to obtain 3 parts: biological process, cell component, and molecular function. GO results showed that the intersection genes were enriched to 498 biological process pathways, 60 cell component expression processes, and 110 molecular function-related processes. A total of 149 KEGG pathways were obtained after KEGG enrichment analysis, mainly in EGFR, MAPK, and PI3K/AKT signaling pathways (**Figures 1E, F**).

### 3.5 Ansofaxine hydrochloride was used to inhibit the progression of hepatocellular carcinoma *in vitro* and *in vivo*

**Figure 2:**
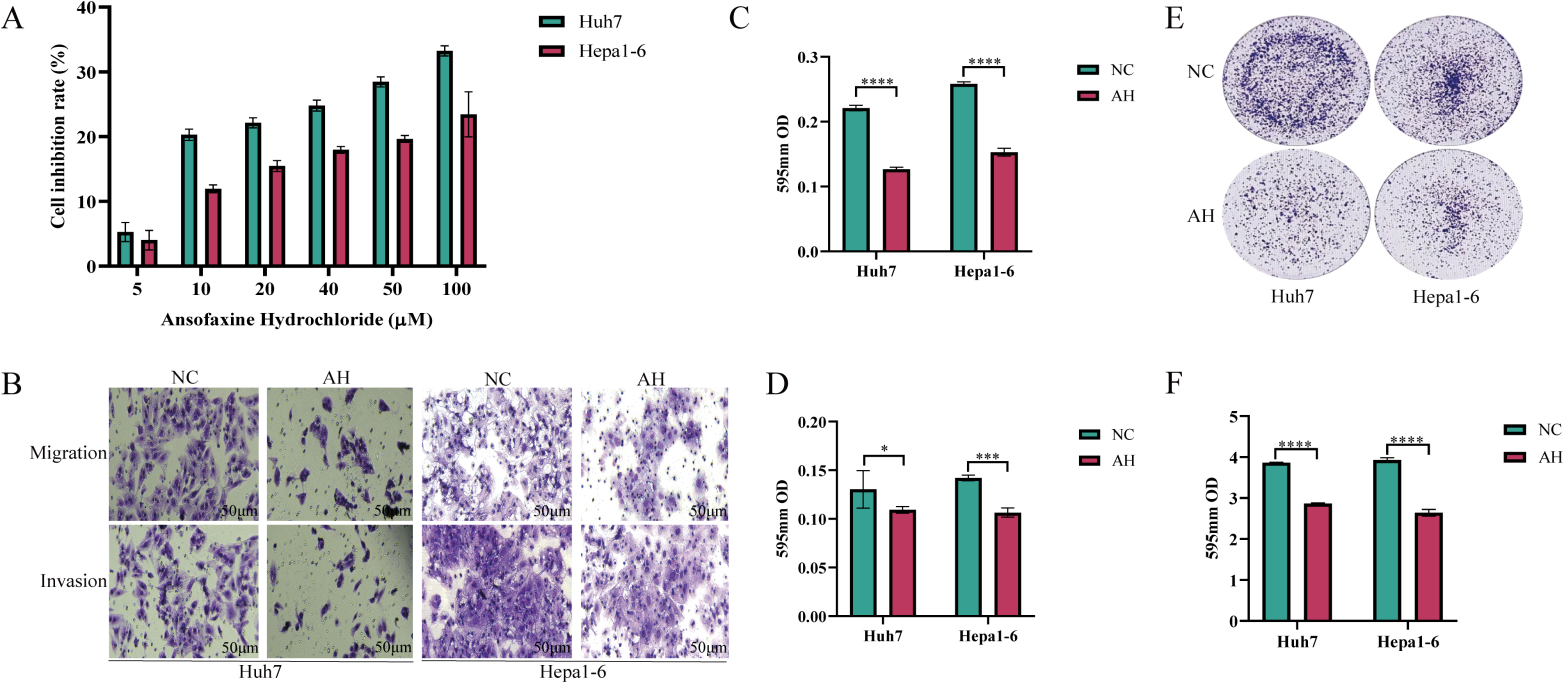
Effects of Ansofaxine hydrochloride on proliferation, plate cloning, migration and invasion of hepatocellular carcinoma cells. **(A)** Effects of different concentrations of Ansofaxine hydrochloride on the proliferation of hepatocellular carcinoma cell lines Huh7 and Hepa1-6. **(B-D)** Effects of Ansofaxine hydrochloride on migration and invasion of hepatocellular carcinoma cell lines Huh7 and Hepa1-6. **(E, F)** Effect of Ansofaxine hydrochloride on clonal formation of hepatoma cell lines Huh7 and Hepa1-6.NC, control group. AH, Ansofaxine hydrochloride group (100μM). *P< 0.05, ***P< 0.001, ****P< 0.0001.

The results of the cell proliferation test showed that Ansofaxine hydrochloride had an inhibitory effect on hepatocellular carcinoma cells with the increase of the concentration. Then we divided the cell function test into two groups: A control group and Ansofaxine hydrochloride group (100μM). To investigate the effects of Ansofaxine hydrochloride on cell invasion, migration, and cloning. The results showed that Ansofaxine hydrochloride significantly inhibited the migration, invasion, and clonogenesis of hepatocellular carcinoma cells (**Figure 2**).

**Figure 3:**
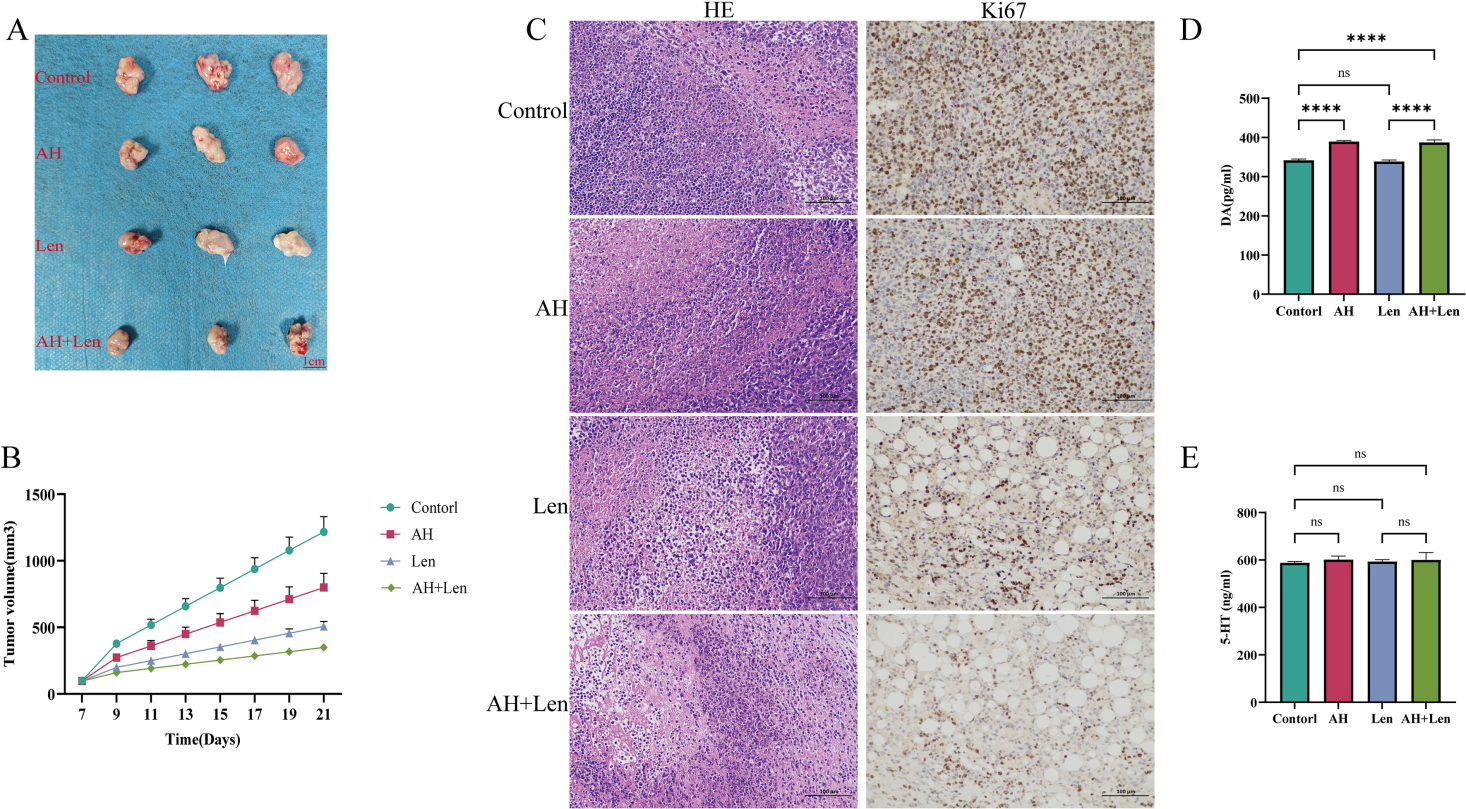
Ansofaxine hydrochloride inhibits tumor growth *in vivo*. **(A, B)** hepatocellular carcinoma tissue, and growth curve. **(C)** HE and Ki67 staining. **(D, E)** peripheral blood DA and 5-HT levels. NC, control group. AH, Ansofaxine hydrochloride group. Len, Lenvatinib group. AH+Len, Ansofaxine hydrochloride, and Lenvatinib group. ns, P > 0.05. ****P< 0.0001.

**Figure 4:**
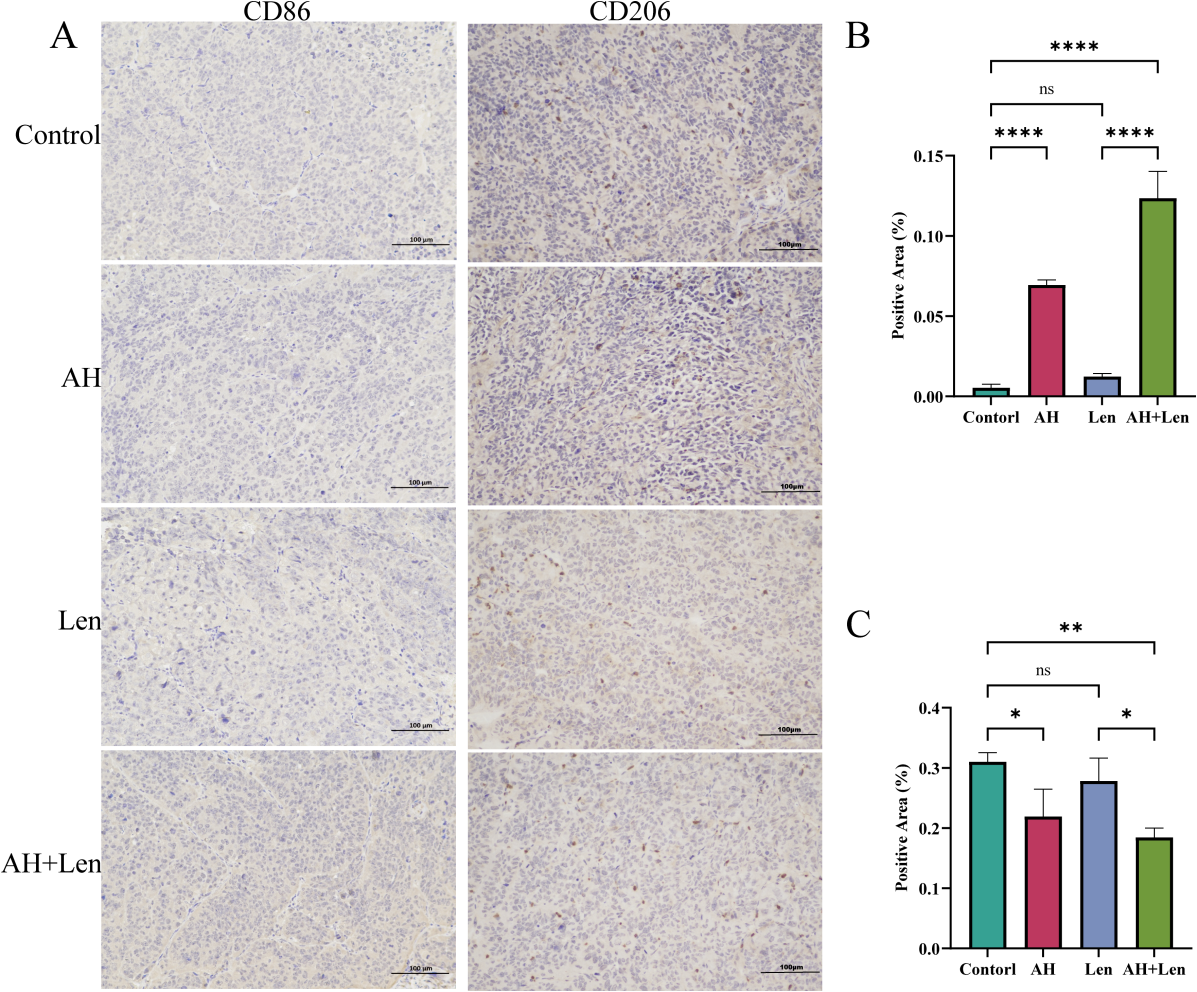
Ansofaxine hydrochloride improves the immune microenvironment of hepatocellular carcinoma tissue *in vivo*. **(A)** hepatocellular carcinoma tissue CD86 and CD206 staining. Positive areas of **(B, C)** CD86, and CD206. NC, control group. AH, Ansofaxine hydrochloride group. Len, Lenvatinib group. AH+Len, Ansofaxine hydrochloride, and Lenvatinib group. ns, P > 0.05. *P< 0.05, **P< 0.01, ****P< 0.0001.

The success rate of the mouse hepatoma xenotransplantation model was 100%. Compared with the control group, Ansofaxine hydrochloride, andLenvatinib hydrochloride can inhibit tumor growth, and the inhibition effect of Ansofaxine hydrochloride is worse than that ofLenvatinib group. However, the combination drug group was superior to the single drug group, and immunohistochemical staining showed that Ki67 was gradually reduced in tumor tissues, especially in the combination drug group. The levels of DA and 5-HT in peripheral blood were significantly higher than those in the control group, suggesting that Ansofaxine hydrochloride inhibits DA and 5-HT uptake (**Figure 3**). In addition, to understand the effect of Ansofaxine hydrochloride on the immune microenvironment of hepatocellular carcinoma, we used CD86 and CD206 to stain, suggesting that M1 macrophages increased while M2 macrophages decreased (**Figure 4**).

**Figure 5:**
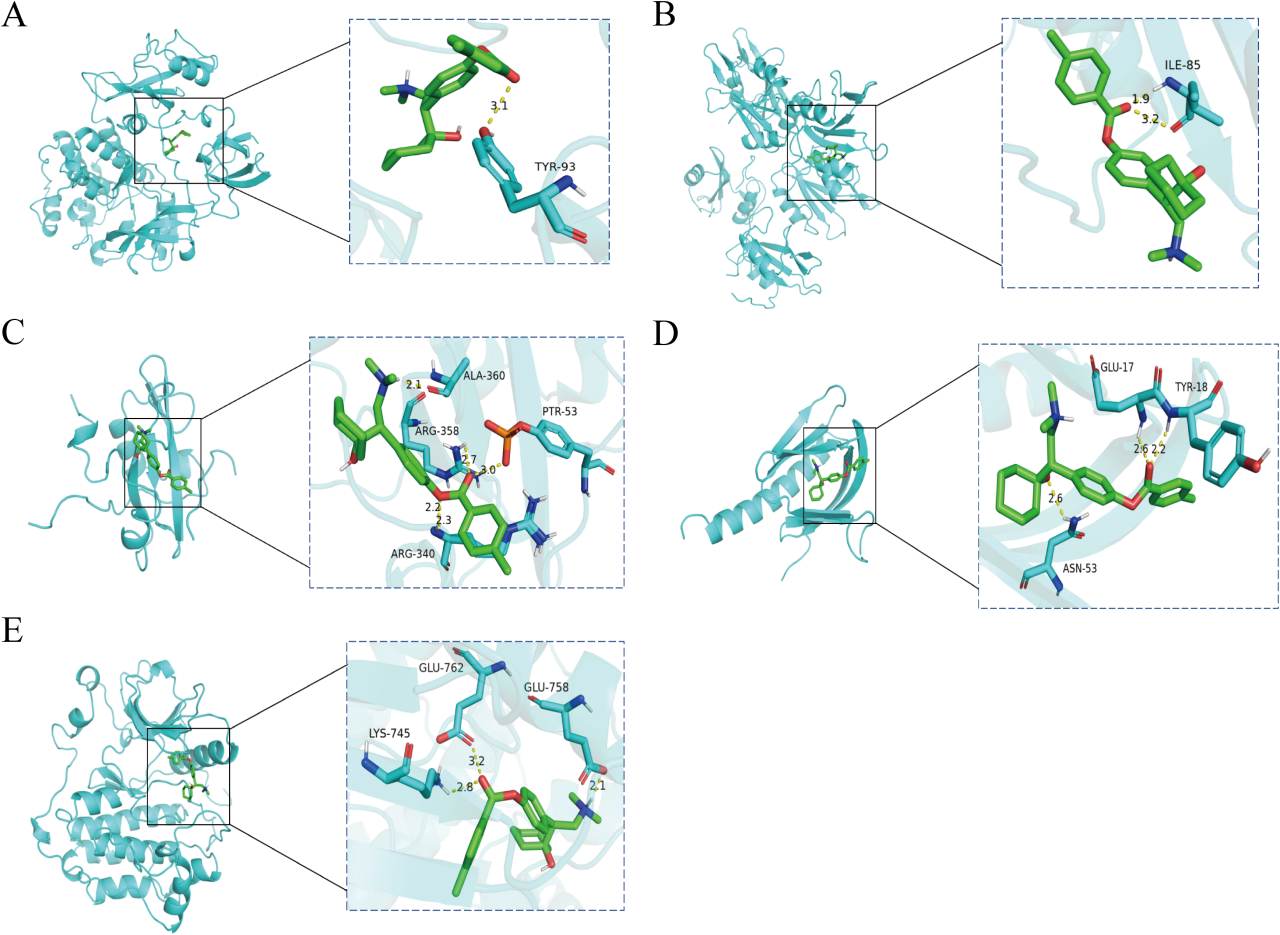
The key gene is docked to a molecule called Ansofaxine hydrochloride. **(A-E)** are SRC, GRB2, PIK3R1, AKT1, and EGFR.

**Figure 6:**
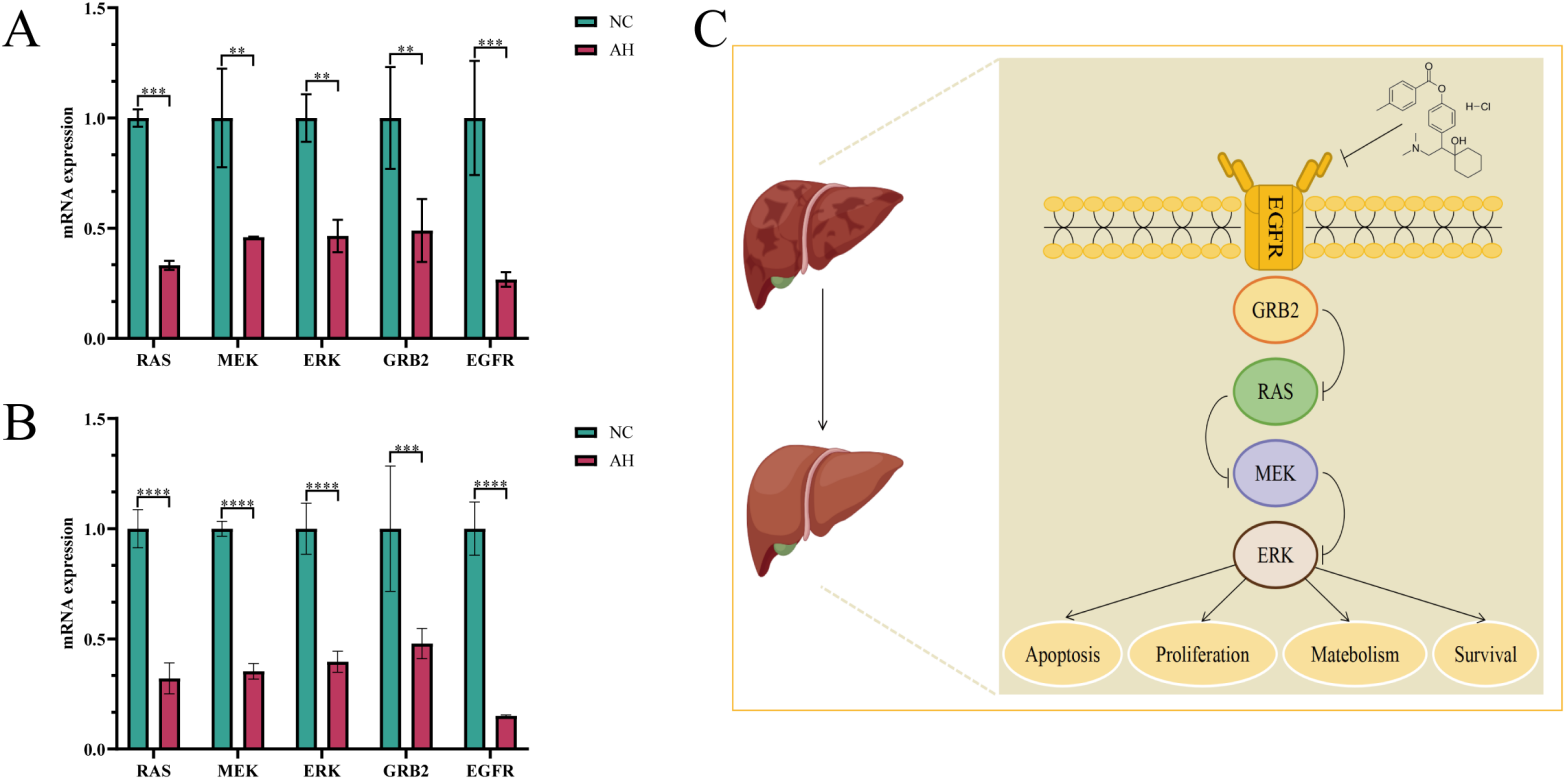
Ansofaxine hydrochloride inhibits the progression of hepatocellular carcinoma through EGFR/MAPK. Detection and analysis of related gene expression levels in Huh7 and Hepa1–6 cells treated with **(A, B)**, Ansofaxine hydrochloride. **(C)** Mechanism diagram of Ansofaxine hydrochloride effect on hepatocarcinoma. NC, control group. AH, Ansofaxine hydrochloride group (100μM). **P< 0.01, ***P< 0.001, ****P< 0.0001.

To explore the key targets and signaling pathways of Ansofaxine hydrochloride for inhibiting hepatocellular carcinoma, we first dock SRC, GRB2, PIK3R1, AKT1, and EGFR, which are in the front of the core genes. Binding energy < -5 kcal/mol indicates good binding activity, and < -7 kcal/mol indicates strong binding activity. The results showed that Ansofaxine hydrochloride binds well to various targets, indicating strong binding activity, and EGFR is a key upstream target gene (**Figure 5**). To verify our conjecture, we further performed PCR experiments. The results showed that Ansofaxine hydrochloride significantly inhibited RAS mRNA expression levels of MEK, ERK, GRB2, and EGFR (**Figure 6**). These results suggest that Ansofaxine hydrochloride may inhibit the progression of hepatocellular carcinoma through EGFR/MAPK.

## 4 Discussion

With the diagnosis of hepatocellular carcinoma and follow-up treatment, patients face the psychological burden of fear of disease, physical pain, and discomfort, resulting in depression. While depression affects the curative effect, it induces the occurrence and progression of hepatocellular carcinoma, and the progression of hepatocellular carcinoma further aggravates the development of depression. The two influence and promote each other. Inflammatory mediators and neurotransmitters are important mechanisms of depression in hepatocellular carcinoma. Cancer-related depression may be associated with pro-inflammatory mediators, mainly through the kynurenine pathway leading to 5-HT reduction, pro-inflammatory mediators impacting glucocorticoids, affecting the hypothalamic-pituitary-adrenal axis, and toxicity through glutamate excitation (21). In addition, there is increasing evidence that 5-HT is involved in a variety of liver lesions, 5-HT promotes the malignant behavior of hepatocellular carcinoma cells, and the 5-HT receptor is highly expressed in HCC patients (22, 23) The three neurotransmitters, 5-HT, dopamine (DA), and norepinephrine (NE), and their metabolites have significant effects on emotional regulation, cognitive performance, and psychological stress response (24). These neurotransmitters regulate the development of depressive symptoms. Our study showed that peripheral blood DA was significantly elevated in mice treated with Ansofaxine hydrochloride, while 5-HT was comparable to untreated levels, which explains part of the effect of Ansofaxine hydrochloride on HCC.

Epidermal growth factor receptor (EGFR) is highly expressed in various solid tumors such as hepatocellular carcinoma, non-small cell lung cancer, and breast cancer, which promotes the development, metastasis, and invasion of hepatocellular carcinoma by enhancing the downstream signal, increasing mutation frequency and activating abnormal bypass, leading to poor prognosis (25–27). Therefore, intervention strategies targeting EGFR and its downstream pathways provide a new direction for the treatment of tumors. In this study, we found that Ansofaxine hydrochloride can interact with EGFR with good binding energy, and subsequent experiments confirmed that Ansofaxine hydrochloride can significantly reduce the gene expression level of EGFR. It is suggested that Ansofaxine hydrochloride may play an antiseptic role by mediating EGFR. Studies have shown that EGFR is the synthetic lethal target of Lenvatinib and is involved in the formation of Lenvatinib resistance through a variety of pathways such as mediated feedback activation of the signal axis (25, 28). Given the inhibitory effect of Ansofaxine hydrochloride on EGFR, we hypothesized that Ansofaxine hydrochloride may synergically enhance the targeting of Lenvatinib. To verify our hypothesis, we combined the two drugs to treat HCC, and the results showed that Ansofaxine hydrochloride can enhance the efficacy of Lenvatinib and is associated with the EGFR pathway. These findings provide a new option for the treatment of hepatocellular carcinoma. In this study, we mainly explored the inhibitory effect of Anshufacine hydrochloride on the growth of hepatoma cells and the EGFR/MAPK pathway. We preliminarily evaluated the effect of its combined use with lenvatinib. However, it is worth noting that this study did not use lenvatinib-resistant strains for the experiment, which, to some extent, limited our in-depth understanding of the efficacy of Anshufacine hydrochloride in the context of drug resistance.

MAPK pathway is key in cell growth, proliferation, differentiation, and apoptosis (29). In hepatocellular carcinoma, the MAPK pathway is often abnormally activated, leading to uncontrolled cell proliferation, enhanced angiogenesis, immune escape, and chemotherapy resistance, and ultimately promoting tumor growth and metastasis (30). Studies have shown that Lenvatinib can inhibit the proliferation, angiogenesis, and invasion of hepatoma cells by inhibiting several key kinases in the MAPK pathway, thus blocking downstream signal transduction, and enhancing anti-tumor immune response (31, 32). Notably, our study shows that Ansofaxine hydrochloride inhibits the MAPK pathway and collaborates with lenvatinib to exert anti-hepatocellular carcinoma effects vivo. Therefore, the combination of Lenvatinib and antidepressants can not only further inhibit the progression of hepatocellular carcinoma, but also improve the quality of life of patients by improving their depressive symptoms, to achieve the dual purpose of enhancing efficacy and prognosis. However, this theoretical hypothesis needs further clinical trials to verify its efficacy and safety.

HCC tumor microenvironment (TME) is composed of a complex involving tumor-associated macrophages (TAM), natural killer cells, and other immune cells (33). TAM is the most abundant matrix component of TME in HCC and plays a role in promoting disease progression by enhancing tumor growth, angiogenesis, and metastasis, as well as checkpoint-blocking immunotherapy resistance to targeted drugs (34, 35). Therefore, selective targeting of immunosuppressive M2 macrophages in TME is expected to play a synergistic role in the current targeted drugs for HCC. Jing Q et al. ‘s study showed that Ansofaxine hydrochloride promotes the proliferation and infiltration of M1, CD8+T, and NK cells, enhances anti-tumor immunity, and inhibits the growth of colon cancer (20). To explore the effect of Ansofaxine hydrochloride on hepatocellular carcinoma macrophages, immunohistochemical staining was performed, and it was found that CD86 was significantly increased after Ansofaxine hydrochloride intervention, while CD206 was significantly down-regulated. It is suggested that Ansofaxine hydrochloride may increase M1 macrophage infiltration and decrease M2 macrophage polarization, indicating that Ansofaxine hydrochloride can improve and enhance anti-tumor immunity and play an anti-tumor role.

Although the experimental data of this study have not directly demonstrated the specific mechanism by which Anshufacine hydrochloride induces apoptosis or autophagy in hepatoma cells, existing studies have shown that various antidepressant drugs can affect the survival and death of tumor cells by regulating the expression of apoptosis-related proteins and autophagy-related genes (14, 36, 37). Therefore, future studies can further explore whether Anshufacine hydrochloride enhances its anti-liver cancer effect by regulating apoptosis and autophagy pathways. Furthermore, this study found that Anshufacine hydrochloride can enhance the anti-tumor immune response by regulating immune cells in the immune microenvironment. Therefore, future studies can further explore the prospects of the combined application of Anshufacine hydrochloride and immune checkpoint inhibitors. Combination medication may produce a stronger anti-liver cancer effect by simultaneously blocking the immune escape pathway and activating the anti-tumor immune response. In addition, the safety and efficacy of combination therapy can also be evaluated through preclinical experiments, providing a theoretical basis and experimental evidence for future clinical trials. Through this combined treatment strategy, it is expected to provide more personalized and effective treatment plans for liver cancer patients.

Finally, it should be noted that there are some limitations to the study. First of all, the animal experiment in this study only carried out the subcutaneous xenograft tumor hepatocellular carcinoma mouse model, and the subsequent experiment needs to adopt a better orthotopic hepatocellular carcinoma mouse model to simulate the tumor microenvironment more truly. Although we observed significant anti-tumor effects, the limitation of the number of mice might have affected the stability and reliability of the results. Secondly, due to the limitation of experimental conditions, only immunohistochemical staining was carried out to explore the immune microenvironment and flow cytometry was also needed to more accurately identify the types and proportions of macrophages.

## 5 Conclusion

Through this study, we preliminarily confirmed that Ansofaxine hydrochloride has the effect of inhibiting hepatocellular carcinoma cell growth and may be closely related to inhibiting EGFR/MAPK. In addition, we found that Ansofaxine hydrochloride also has the effect of increasing M1 macrophages and inhibiting M2 macrophages. The combination of Ansofaxine hydrochloride with Lenvatinib may enhance the efficacy of HCC patients with concomitant depression.

## Data Availability

The raw data supporting the conclusions of this article will be made available by the authors, without undue reservation.
